# Too advanced for assessment? Advanced materials, nanomedicine and the environment

**DOI:** 10.1186/s12302-022-00647-7

**Published:** 2022-08-16

**Authors:** Silvia Berkner, Kathrin Schwirn, Doris Voelker

**Affiliations:** German Environment Agency, IV2.2 Pharmaceuticals and Nanomaterials, Woerlitzer Platz 1, 06844 Dessau-Roßlau, Germany

**Keywords:** Nanomedicine, Medicinal advanced materials, Nanopharmaceuticals, Safety assessment, Environmental impact, One Health

## Abstract

Advanced materials, and nanomaterials, are promising for healthcare applications and are in particular in the spotlight of medical innovation since rapidly developed nano-formulated vaccines provide relief in the SARS-CoV-2 pandemic. Further increased rapid growth is to be expected as more and more products are in development and reach the market, beneficial for human health. However, the human body is not a dead end and these products are likely to enter the environment, whereas their fate and effects in the environment are unknown. This part of the life-cycle of advanced medicinal products tends to be overlooked, if the perspective is human-centered and excludes the connectedness of human activity with, and consequences for our environment. Gaps are reviewed that exist in awareness, perspective taking, inclusion of environmental concerns into research and product development and also in available methodologies and regulatory guidance. To bridge these gaps, possible ways forward start to emerge, that could help to find a more integrative way of assessing human and environmental safety for advanced material medicinal products and nanomedicines.

## Introduction

**Fig. 1:**
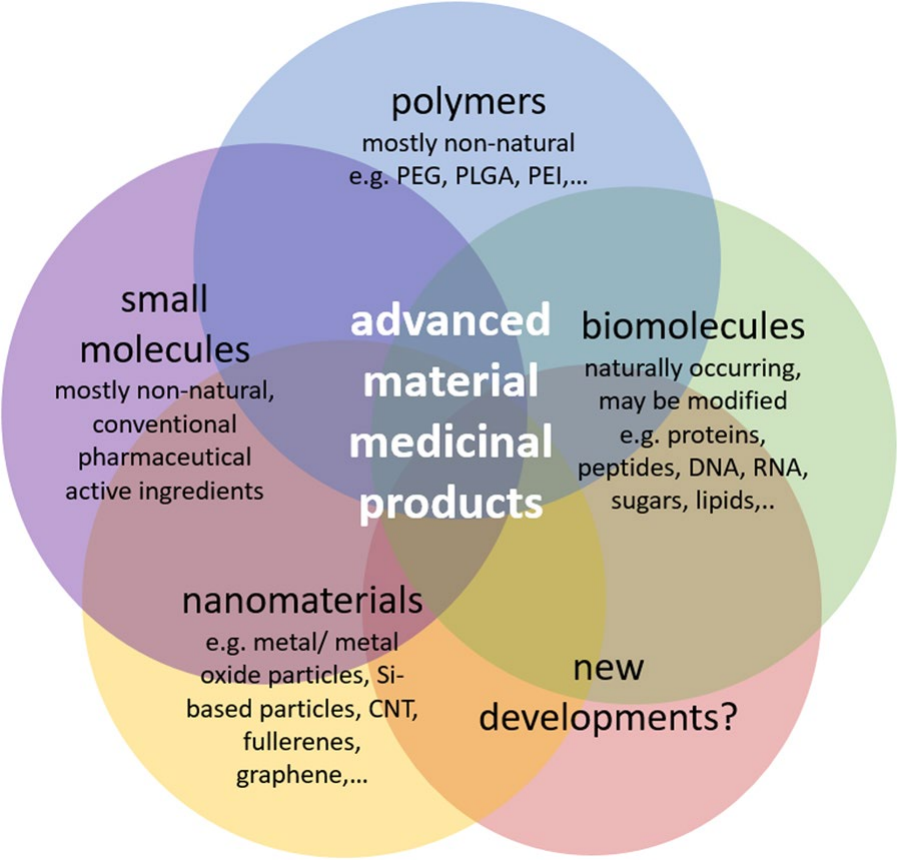
Overview on building blocks of advanced material medicinal products. *PEG* polyethylene glycol, *PLGA* poly(lactic-co-glycolic acid), *PEI* polyethyleneimine, *CNT* carbon nanotubes

The term “advanced materials” describes materials that are rationally designed in order to fulfill the functional requirements of a certain application [1] with novel or enhanced properties that improve performance over conventional products [2]. The term is often used for more complex combinations of different components or building blocks to obtain materials with specific properties and functions (see Fig. 1). The term overlaps with “nanomedicines” or “nanopharmaceuticals” but is more inclusive. The past years have seen a rapid increase in research and development of medicinal products and devices based on advanced materials [3] [2]. At the same time, the number of products with medical applications based on advanced materials that reach the market is increasing rapidly [4]. The current pandemic situation further acts as a catalyst to speed up the development and highlight benefits of novel treatments based on advanced materials like nanobiomaterials. This is exemplified by the development and successful marketing authorization at unprecedented speed of nano-formulated vaccines based on modified RNA to fight the SARS-CoV-2 pandemic [5, 6]. Thus, environmental exposure, due to the increasing use of advanced materials in biomedical applications, “has become inevitable” [7, 8]. Therefore, it is time to take a look at gaps that might exist on different levels concerning the environmental assessment for advanced material medicinal products as well as ways forward helpful to address the identified gaps.

## Gaps...

### Awareness and perspective taking

In a study that collected expert perspectives on potential environmental risks from nanomedicines and adequacy of the current guideline on environmental risk assessment [9], Mahapatra et al. [10] concluded that “very few studies have explored the environmental risks from nanomedicine, especially none on expert's perceptions on environmental risks from nanomedicine […] The instinctive and spontaneous discussion on possible human health risks from nanomedicine shows that the concept of environmental risk assessment seems to be distant and distinct (except for specialist eco-toxicologists). […] our research highlights a significant gap in terms of awareness of environmental regulations as well as a lack of orientation towards an ecosystem perspective.”

### Legislation

This lack of awareness and a wider perspective including the environment, is in contrast to other fields. An example is REACH, a comprehensive system concerned with human and environmental safety of chemicals in the EU [11]. It has been amended in 2018 to include the assessment of nanomaterials [12] and provides information requirements specific for nanomaterials. However, pharmaceuticals are exempt from REACH obligations [13]. On a global scale, they are also exempt from the Globally Harmonized System (GHS) dealing with classification and labeling of chemicals worldwide [14]. Pharmaceutical-specific regulatory frameworks exist, e.g., in the EU and North America, that include an assessment of the environmental impact into the marketing authorization procedure for medicinal products [9, 15]. For advanced materials, however, it should be noted that they may fall within different regulatory frameworks differing in requirements. In the EU, on the one hand, medical devices—which are developing into one of the major application fields for advanced materials—do not require any environmental impact or risk assessment [16].

### Regulatory guidelines

On the other hand, for human medicinal products an environmental risk assessment framework has been established in 2006. The assessment is based on the “Guideline on the Environmental Risk Assessment for Medicinal Products for Human Use” issued by the European Medicines Agency (EMA), shortly named EMA Guideline in the following [9]. The framework is tame already for conventional pharmaceuticals, as the outcome of an environmental assessment is not considered in the risk–benefit balance and thus does not influence the granting of a marketing authorization. Consequently, the timely and complete submission of environmental assessment dossiers, study reports and also the quality of underlying studies are impacted [17]. Additionally, results and data from studies, though in principle not confidential, are not or only to a very limited degree publicly accessible [18].

In addition, the EMA Guideline includes exemption clauses, which could potentially lead to advanced material medicinal products falling through the cracks. One example is vaccines, which are exempted from providing an assessment of fate and effects in the environment and a simple justification for the absence of an ERA is considered adequate [9]. The EMA Guideline is currently under revision and the revised draft version [19] states that “Vaccines are unlikely to result in a risk to the environment and the ERA may consist of a justification for not submitting ERA studies”. Vaccines, as presently very tangible in the current pandemic situation, are administered to a large population in a short period of time. This implies that exposure from vaccine components, if occurring to the environment, will be on a large scale, which is in contrast to a lack of information on environmental exposure, fate or effects. In the last years, “there has been an explosion of nanomaterials explored as new vaccines” as Fries et al. [20] observed. Nanomaterials or advanced materials might be used as vaccines or as excipients, like adjuvants. One example for the latter is fullerenol (surface modified C_60_-fullerene), that has been described as an adjuvant for vaccines [21] and has been shown to have ecotoxicological effects on a number of species, ranging from antimicrobial activity [21] to effects in plants and aquatic species [22]. Polymers can also be used as delivery vehicles for vaccines. For example, fluoropolymers have been described as delivery vehicles for anti-cancer vaccines. Fluorinated F7- and F13-polyethyleneimines (PEI) have been found to be promising delivery agents for antigens to the cytosol of dendritic cells [23]. The transformation products that are formed when these polymers degrade in the environment are perfluorocarboxylic acids and other related perfluorinated compounds [24]. These substances are of very high environmental concern because they combine the undesirable properties of being persistent, bioaccumulative and toxic (PBT). Perfluorooctanoic acid has been identified as persistent organic pollutant (POP) according to the Stockholm Convention [25]. The Convention covers chemicals that are considered so hazardous for the environment and human health due to their tendency to persist and accumulate in organisms, that their use is being restricted worldwide. For the whole group of per- and polyfluorinated alkylated substances (PFAS), to which also perfluoroalkanoic acids belong, a restriction is proposed under REACH for all but essential uses [26, 27], which however would exempt medical uses.

### Fate and effects testing

The EMA Guideline also exempts products containing amino acids, proteins, peptides, carbohydrates, and lipids from providing a study-based environmental assessment [9]. However, amino acids, peptides, proteins, lipids and carbohydrates may be part of an advanced material therapeutic agent, e.g., as a building block together with other components, that might actually require more attention concerning environmental fate and effects. Examples are antibody drug conjugates (ADC) that are successfully used in cancer treatment. They consist of three building blocks: (i) a cytotoxic molecule, like, e.g., monomethyl auristatin E (MMAE) which is coupled via (ii) a linker with a cleavable moiety and in some cases including additionally moieties to, e.g., improve solubility (like polyethylene glycol (PEG)) to (iii) an antibody (fragment) for targeted delivery to cancer cells, like, e.g., brentuximab vedotin [28]. This ADC may be considered a simple example for an advanced material medicinal product, nevertheless represents a combination of different building blocks combined to obtain new functionalities. It illustrates how easy advanced material medicinal products may slip through regulatory gaps. If the complete molecule, like in our example brentuximab vedotin, is tested, e.g., for ready biodegradability (the first step in environmental fate testing according to the EMA Guideline [9]), the outcome will be dominated by the large protein part and the whole molecule will be classified as readily biodegradable and thus without concern for the environment. The large protein moiety, however, masks the fate of the cytotoxic molecule MMAE. This part of the active ingredient is not readily biodegradable and thus may reach the environment. Effects on environmental organisms are likely due to its high cell toxicity that actually prevents its use in free form in humans [28]. This illustrates the need to consider the building blocks as well as the whole entity for fate and effect testing. Additionally, this example shows, that with more sophisticated delivery technologies, even more potent molecules may be used in advanced pharmaceutical and therapeutic agents.

Although in the case of ADC, the biomolecule part of the active ingredient may be of no environmental concern, an exemption for products containing amino acids, proteins, peptides, carbohydrates, and lipids [9] may not be warranted for other advanced medicinal products and nanomedicines based on biomolecules like, e.g., peptides and nucleic acids. Naturally occurring biomolecules often are highly beneficial for therapeutic purposes, however are not stable and/or their bioavailability is not sufficient for medical uses, especially for oral delivery. Therefore, naturally occurring compounds are often modified to enhance stability and increase bioavailability [29]. There is a wide spectrum of modifications ranging from replacing or modifying single or multiple amino acids or nucleotides with natural or non-natural analogues to attaching further molecules, or backbone modifications. Examples are modified peptides or modified DNA or RNA molecules. There are already products on the market, e.g., patisiran [30], an siRNA formulated as lipid nanoparticles, or givosiran [31], a phosphorothioate modified siRNA active ingredient. It is presently unclear, how to assess the environmental impact of such compounds. Environmental relevance cannot be excluded per se, as nucleic acids can be taken up by and affect environmental organisms [32]. Also, for peptides, which may be excreted in intact form [33], it has already been shown that exogenous peptides can be taken up by fish, e.g., for GnRH peptides from water [34].

There are clearly knowledge gaps concerning the environmental fate and effects and regulatory assessment of these (modified) biomolecules [35], which can be considered nanomedicines, advanced material medicinal products or constitute building blocks for such compounds. Knowledge gaps are enormous when considering the whole range of advanced therapeutic agents under development: products that act based on morphological changes like, e.g., so-called nanotransformers [36], DNA origami scissors [37] or nanocarrier systems including nanomaterials like, e.g., graphene- and CNT (carbon nanotube)-based products [38]. Information and ways to characterize and assess environmental fate and effects of such functionally novel compounds, but also for polymers and other carrier systems, or diagnostics [39] are lacking.

### Exposure estimation

There are also gaps concerning exposure estimations for advanced material medicinal products. For human pharmaceutical environmental assessment in the EU, a mass-based action limit [9] is used, that is applied in a product-specific way. Thus, already for small molecule pharmaceuticals an approach based on active ingredients is lacking, as one active is often marketed in different products. For advanced material products, it has additionally to be considered that the same building blocks (e.g., the cytotoxic molecules in ADCs or other carrier-based anti-cancer treatments, or recurring molecules in carrier building blocks) can be part of different products and also of different active ingredients. There is a gap in accounting for this additivity in exposure estimations. Additionally, new products often enter the market via orphan applications or with a narrow indication range, which stops an environmental assessment due to low exposure assumptions. Normally the first application for marketing authorization is seen as the time point to request in-depth information from the applicant. In later applications for the same active ingredient (e.g., due to the addition of further indications, which leads to higher exposure estimates), it is not considered adequate to ask for in-depth information, as concerns should already have been addressed in the first application.

Environmental exposure may occur by excretion from patients. This points to the crucial role of ADME (absorption, distribution, metabolism, elimination/excretion) studies for the environmental assessment of complex advanced material active ingredients. From a human-centered point of view, excretion studies focus on proving that a certain substance is excreted from the patient body, but not necessarily in which form or quantitative amount. Therefore, quantitative data for excretion via urine and feces may not be available, which are parameters very helpful for an environmental assessment (see e.g., the published information by EMA (only qualitative and not quantitative) for polatuzumab vedotin, another ADC on the market [40]). A contributing factor might be the ICH (International Council for Harmonisation of Technical Requirements for Pharmaceuticals for Human Use) Guideline S6 on preclinical safety evaluation of biotechnology-derived pharmaceuticals discouraging mass balance studies [41], which might lead to not reporting excretion data for products containing a biomolecule as a building block.

These examples highlight that there are knowledge gaps in how to address characterization, metabolism, excretion and environmental exposure, fate and effects for advanced medicinal products. These knowledge gaps concern basic research and also the application of scientific findings in more standardized procedures usable for regulatory assessment.

In summary, advanced material medicinal products and nanomedicines are rapidly developing, with an increasing number of products reaching the market and being administered in some cases to large parts of the population in short time periods. All the while there are a lot of gaps on different levels in awareness, perspective taking, transparency, legislation, regulatory guidance and basic research as well as more applied methodological research.

## ... and possible ways forward

### Basic research and methodological guidance

To address the illustrated gaps, some possible ways forward are emerging. Research is starting to address knowledge gaps, e.g., novel endpoints in ecotoxicological testing like immunotoxicology are considered [42], as well as effects based on physical interactions [43]. The fate of water-soluble polymers in the environment is starting to be addressed by monitoring, e.g., for PEG [44]. These are starting points for more urgently needed research to close knowledge gaps on how to characterize and monitor advanced materials concerning physical–chemical properties, exposure, fate and effects in the environment. EMA has identified this area in their publication on regulatory science research needs [45]. Existing assessment schemes seem basically applicable, however often adaptations or in some cases, new testing strategies are required [46, 47]. Research calls that fund applied studies concerning environmental fate and effects of compounds or building blocks used for advanced material medicinal products and nanomedicines are helpful to support otherwise neglected applied research. An example is Biorima [48], a project concerned with nanobiomaterials used in advanced therapy medicinal products and medical devices. Some case study results from this project on environmental effects of rather simple nanomaterials have already been published [49]. Also, on a more applied level for testing for environmental impacts, the test guidelines (TG) and guidance documents (GD) developed in the framework of the OECD test guideline program are valuable tools for standardized, mutually acceptable assessments. Over the last 10 years, several new TG and GD for nanomaterials have been developed and adopted, or are in the process leading to adoption [50]. For environmental testing, besides ecotoxicity tests, which are more straightforward to adapt for nanomaterials, a special focus of the mentioned activities is on environmental fate [51]. Concerning ADME information, a project on a new nanospecific test guideline for toxicokinetics is underway [52]. However, the focus is on determining internal exposure of test animals. Thus, for advanced material medicines there is still a need for guidance that specifically addresses the questions if, in which amount, and in which form the active substance or their building blocks or metabolites are excreted from patients.

Information is accumulating that nanobiomaterials are excreted. Hauser and Nowack [53] conducted a meta-analysis of publicly available pharmacokinetic studies for 192 nanobiomaterials which are representative for products on the market [4, 54]. For 82% of the materials total excretion was equal to or above 10% of the administered dose, irrespective of administration route. Especially for orally administered nanobiomaterials high excretion was observed, most frequently in the range of 80–100% of the administered dose. These findings underline the importance to consider environmental exposure, fate and effects when researching, developing, authorizing and marketing of advanced materials in medical applications. Thus, assessing environmental impacts may require information from the non-clinical safety ADME studies. In recent years a number of initiatives has started to develop guidance for marketing authorization of advanced materials with medical applications, e.g., the International Pharmaceutical Regulators Programme (IPRP) Nanomedicines Working Group [55]. Considering the full life-cycle of a medicinal product, including data needed for assessing the relevant compounds or metabolites for testing fate and effects in the environment, would be very helpful at this stage. New approaches might be needed as it has been observed that nanoparticles pharmacokinetic behavior differs from that of small molecule pharmaceuticals [39].

#### Regulatory guidelines

There are also developments in the field of regulatory guidelines. Currently, EMA is revising its environmental assessment guideline for human medicinal products. The published draft version [19] does not mention advanced material or nanomedicine products. It is time to consider this rapidly growing field and develop advice, as the standard assessment approach might not be suitable without adaptations and individual advanced materials may require different approaches. Exception clauses, as described above, have been identified as potentially causing gaps in the environmental part of safety assessment. Therefore, it should be considered to refrain from using exceptions from providing a data-based environmental assessment, as this seems no longer timely considering the examples given for emerging advanced material medicinal products (e.g., vaccines) and the rapidly growing capabilities to modify or engineer even natural substances produced by organisms, e.g., as recently described for proteins [56]. Nevertheless, it might be important to recognize, that due to the wide variety and complexity of advanced materials in medical applications, which only starts to emerge and cannot be fully foreseen yet, a one-size-fits-all strategy might be elusive. A modular approach could be valuable in offering guidance for specific components or building blocks of advanced material or nanomedicinal products (see Fig. 1), i.e., not to have to “group” a product into a certain category, but to base assessments on the building blocks or components that constitute the product. An approach that overcomes the drawbacks of product-specific assessment and takes into consideration “building block additivity” for exposure estimation would be helpful. For other regulatory frameworks, guidance has been proposed, e.g., for human health for nano- and advanced material pesticides [57] while gaps have been identified for the environmental assessment [58]. Nanomaterial-specific guidance for environmental and health assessment has been published for food and feed [59, 60]. These examples could provide input for the field of pharmaceuticals and medical products.

#### Legislation

On the legislative level, the EU Commission has started the process of reviewing human pharmaceutical legislation with the Pharmaceutical Strategy for Europe [61]. This process might offer a way forward to strengthen the part of the marketing authorizations concerning the environmental impact in general, and to recognize the need for an adequate assessment of nanomedicines/advanced material medicinal products. Also, on a global level, inclusion of substances used in medicinal products into GHS would be a valuable step forward, especially considering regions of the world which have not yet developed specific regulatory systems. Also, the inclusive approach on human and environmental safety aspects taken by the recent EU Regulation on veterinary medicinal products [62] that specifically addresses considerations for nanomedicines and RNA-based medicines [63] could serve as helpful example.

#### Awareness, perspective taking

As especially fostering exchange and understanding between different “worlds” of safety assessors—environmental and human—is needed [10], it would be helpful to install a permanent working group at EMA for human pharmaceuticals. This group could discuss topics related to the environmental assessment of medicinal products involving more experts in the field of environmental exposure, fate and effects. Such a group already exists at EMA for veterinary medicinal products and has proven very helpful to develop guidance. However, regulatory involvement is only the last step towards marketing a medicinal product. Safe and sustainable by design (SSbD) principles, as included in the EU Chemical Strategy for Sustainability [64], can guide scientists and product developers to include these considerations already earlier in the research and development process [65, 66], although this has been pointed out to be difficult for nanomedicine [67]. Considering SSbD is not meant to limit exploring promising new candidates for development, but to involve experts from different fields, to be able to identify concerns from inter alia an environmental perspective at an earlier point in the development process. The example given above for the fluorinated PEI polymers highlights the need for an early involvement of environmental experts to help to identify environmental implications already in the development stage and to explore possible alternatives or develop risk mitigation strategies. In nanomedicine, biopersistence has been recognized as a problem for human safety. Two ways forward have been proposed: (i) using biodegradable (nano-)materials and (ii) using ultrasmall nanoparticles (< 10 nm), which are rapidly eliminated by glomerular filtration [39]. From an environmental perspective, the first approach seems to be worth exploring, as in the best case, exposure of the environment could be avoided or at least reduced. The second approach would lead to high rates of excretion from the patient body leading to exposure of the environment. Yet, it needs to be kept in mind, that physiological biodegradability and harmlessness cannot automatically be translated into the same characteristics in the environment.

#### Data availability, accessibility, and quality

In that regard, also data availability and accessibility are crucial. This includes recommendations for reporting research outcomes, as e.g., described in the MIRIBEL standard (minimal information reporting in bio-nano experimental literature, [68]). However, also in this case it is important to include the wider perspective and consider potential fate and effects in the environment after treatment of patients, as highlighted by comments on the proposed MIRIBEL criteria from Hansen and colleagues [69]. They recommend to also consider the NanoCRED reporting checklist [70] and the work of the OECD on nanomaterials to improve transparency and reproducibility within nanobiomedicine, which could provide a starting point also for other advanced material medical products. The FAIR (findable, accessible, interoperable and reusable) data principles [71] are also recommended for nanotechnology data and recently an interface for human and environmental nanosafety data has been described [72]. Also, data on composition, physical–chemical characterization, metabolism, excretion and environmental exposure, fate and effects gained from marketing authorization procedures can provide important information for further regulatory purposes and research. Therefore, improvements on data accessibility for studies conducted in a regulatory context are needed, as outcomes from marketing authorization procedures concerning environmental exposure, fate and effects are presently not or very difficult to access [18] and the FAIR principles might also offer orientation for dealing with these data in a more transparent and sustainable way.

## Summary and outlook

**Fig. 2:**
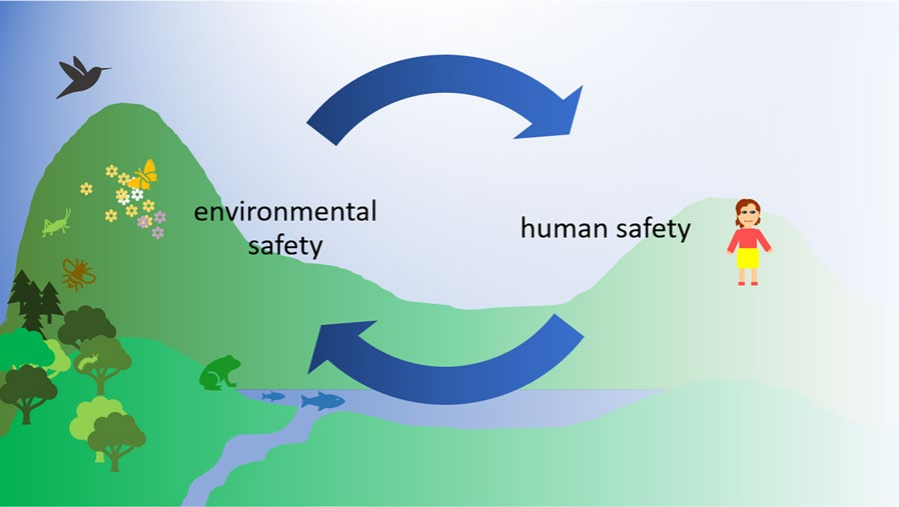
Illustration of the One Health principle for safety assessment: human safety and environmental safety are interlinked, there is no human health without environmental health

Advanced therapeutics and nanomedicines might actually be part of the solution to a more sustainable approach to pharmacological treatment. For instance, increased oral bioavailability or prolonged half-life for orally administered nanomedicines have been successful in reducing dosage, frequency of administration, and toxicity, for e.g., chemotherapeutic agents [73, 74]. This might also be helpful to reduce harmful consequences for the environment, which are observed for small molecule pharmaceuticals [75–77]. At the same time, it is important to also consider potential environmental impacts from advanced material medicinal products or nanomedicines. There are considerable gaps in research and knowledge both basic and applied. In some cases, existing methodological and regulatory guidance may be useful, however require adaptations. A modular approach considering the different building blocks and the whole entity might be a helpful way forward to address regulatory assessment. The safety assessment for advanced therapeutics and nanomedicines can benefit from cooperation and learning across different disciplines [10], embracing the SSbD principles, and from including different perspectives as exemplified in the One Health principles [78], as illustrated for safety assessment in Fig. 2. Human and environmental health are interlinked. Presently, the risk–benefit assessment for human pharmaceuticals does not include any considerations outside the treated patient. This principle clearly has advantages in reducing complexity. At the same time, it seems timely to also consider the context and the more complex environment of which also humans are a part and their interactions and connectedness with the environment.

A more inclusive One Health approach serves well in addressing other pressing issues like antibiotic resistance [79] and is also considered for advanced veterinary medicinal products [80]. These principles could also serve as valuable input for addressing the safety of human advanced material medicinal products and nanomedicines.

## Data Availability

Not applicable.
